# “Am I doing everything right enough?”: a mixed methods evaluation of the early post-bariatric surgery period

**DOI:** 10.1186/s40359-026-05225-7

**Published:** 2026-08-06

**Authors:** Sevgi Koroglu Gokbel, Nilgün Sert Baş, Gulgun Durat, Dilek Aygin, Adem Yüksel, Kerem Karaman

**Affiliations:** 1 Department of Nursing, Faculty of Health Sciences, Sakarya University, Kemalpaşa, Esentepe Campus, Üniversite Cd., Serdivan, Sakarya, 54050 Turkey; 2 Department of Nursing, Institute of Health Sciences, Sakarya University, Sakarya, Turkey; 3 Department of Surgical Diseases Nursing, Faculty of Health Sciences, Ankara Medipol University, Ankara, Turkey; 4 Department of General Surgery, Faculty of Medicine, Sakarya University, Sakarya, Turkey

**Keywords:** Obesity, Bariatric surgery, Emotion regulation, Personality assessment, Quality of life

## Abstract

**Background:**

The post-bariatric surgery period is a multidimensional experience encompassing individuals’ physical, psychological, and social adjustment processes. During this period, personality functioning and emotion regulation abilities may significantly influence postoperative adjustment and quality of life. Therefore, it is important to address the issue from a holistic perspective. This study aimed to examine the relationship between personality functioning, emotion regulation difficulties, and quality of life after bariatric surgery, and to explore patients’ postoperative experiences using a mixed-methods approach.

**Materials and methods:**

This study was conducted using a concurrent parallel mixed-method design. Quantitative and qualitative data were collected from individuals within the first year after bariatric surgery. In the quantitative phase, 95 participants completed the Inventory of Personality Organization (IPO-TR), the Difficulties in Emotion Regulation Scale–Short Form (DERS-16), and the Post-Bariatric Surgery Quality of Life Scale (PBSQLS). Regression analyses were conducted to examine the relationships among variables. From among the quantitative participants, 18 patients were selected through purposive sampling based on different follow-up months and gender, and participated in semi-structured interviews. Qualitative data were analyzed using Braun and Clarke’s thematic analysis. Quantitative and qualitative data were analyzed separately and then integrated through a joint display approach.

**Results:**

Quantitative analyses indicated that IPO-TR scores strongly and positively predicted PBSQLS–Complications scores and negatively predicted PBSQLS–Biopsychosocial Area scores. When DERS-16 was added to the model including IPO-TR, DERS-16 was not independently associated with PBSQLS–Complications subscale scores and showed a marginally significant association with the PBSQLS–Biopsychosocial Area subscale. Female sex and earlier postoperative follow-up periods (1, 3, and 6 months) were associated with higher scores on the PBSQLS–Complications subscale. Qualitative themes revealed psychological distress and functional difficulties during the 1st–3rd postoperative months; increased body satisfaction and self-confidence during the 6th–12th months; support as well as jealousy/tension in interpersonal relationships; and patterns of externalization, self-criticism, and suspiciousness in the adjustment process. Integration findings indicated that the statistical associations between IPO-TR and the PBSQLS subscale scores were contextualized by the qualitative findings. Quantitative findings related to emotion regulation were further informed by the contextual insights obtained from the qualitative data.

**Conclusion:**

Quality of life after bariatric surgery appears to be associated not only with physical recovery but also with personality functioning and psychosocial processes. The findings highlight the importance of considering psychological vulnerability and adjustment difficulties in early postoperative clinical follow-up. Given the cross-sectional design and the reliance on self-report measures, the findings should not be interpreted as indicating causal relationships. Future studies with larger samples, more comprehensive clinical variables, and longitudinal designs are needed to validate these findings.

**Supplementary Information:**

The online version contains supplementary material available at https://doi.org/10.1186/s40359-026-05225-7.

## Background

Obesity is defined by the World Health Organization (WHO) as excessive fat accumulation that may impair health. It is also a chronic and complex disease that can increase the risk of conditions such as type 2 diabetes and heart disease and negatively affect quality of life [1]. Obesity affects more than 890 million adults worldwide (13%), and this number is projected to reach 1.02 billion (18% of adults) by 2030 [2]. According to the WHO 2022 European Regional Obesity Report, Türkiye has the highest prevalence of obesity in Europe [3].

In the treatment of obesity, surgical methods are recommended in cases where lifestyle modifications such as diet, exercise, cognitive behavioral therapy, and appetite-suppressing medications are insufficient. The American Society for Metabolic and Bariatric Surgery states that bariatric surgery is the most effective method for resolving metabolic diseases and treating obesity [4]. Weight loss following bariatric surgery—or failure to achieve weight loss—affects patients’ quality of life [5]. Assessing quality of life enables monitoring of the impact of postoperative weight loss on daily functioning across physical, psychological, and social domains and allows for a holistic evaluation of the patient [6].

Quality of life, an indicator of surgical effectiveness, is influenced by psychological and emotional factors. The literature has shown that a history of psychiatric disorders, personality traits, depressive symptoms, anxiety, and mood disorders affect quality of life [6, 7]. One of the main underlying causes of maladaptive eating behaviors observed in bariatric surgery candidates is difficulty in emotion regulation. Emotion regulation difficulties refer to problems in recognizing, understanding, expressing, and managing emotional experiences [8]. In this context, the process of coping with and adapting to changes after bariatric surgery may be influenced by individuals’ emotion regulation abilities.

Psychological characteristics related to personality, such as self-management, effortful control, neuroticism, reward sensitivity, and locus of control, have been reported to play an important role in postoperative weight loss following bariatric surgery [9, 10]. One of these characteristics, personality functioning, has been conceptualized within theoretical approaches that describe dimensions of psychological functioning, including identity integration, defense mechanisms, and reality testing. One such approach is Kernberg’s model of personality organization [11, 12]. According to this model, personality organization is conceptualized based on identity integration, the quality of defense mechanisms, and the capacity for reality testing, distinguishing neurotic, borderline, and psychotic levels of personality organization [13].

According to Kernberg’s model, neurotic personality organization is characterized by an integrated identity, more mature defense mechanisms, and preserved reality testing, whereas borderline personality organization is characterized by difficulties in identity integration, more frequent use of primitive defense mechanisms, and impairments in reality testing under conditions of intense stress [13]. Psychotic personality organization, in contrast, is associated with marked disturbances in identity integration, predominant use of primitive defense mechanisms, and severe impairment in reality testing [13].

Theoretically, the different levels of personality organization described by Kernberg are proposed to be associated with individual differences in coping with stress, emotion regulation, and interpersonal functioning [13–18]. Accordingly, Kernberg’s model of personality organization has been considered one of the theoretical frameworks that may contribute to understanding psychological adjustment in chronic illness. Nevertheless, the nature of the relationship between personality functioning and quality of life after bariatric surgery, as well as the mechanisms underlying this relationship, remains insufficiently understood [14–18].

Therefore, further investigation of the relationship between personality functioning and quality of life following bariatric surgery is warranted. However, the existing literature provides limited and inconsistent evidence regarding the nature of this relationship and the psychological mechanisms that may underlie it. Furthermore, although psychological adjustment and quality of life after bariatric surgery have been widely investigated, relatively few mixed-methods studies have examined self-reported personality functioning together with patients’ postoperative experiences. Therefore, the aim of this concurrent parallel mixed-methods study was to quantitatively examine the relationships among personality functioning, emotion regulation difficulties, and quality of life following bariatric surgery, and to qualitatively explore patients’ postoperative experiences. In addition, the study aimed to achieve a more comprehensive understanding of these relationships through the integration of quantitative and qualitative findings. The research questions are as follows:

1. Are personality functioning and emotion regulation difficulties associated with quality of life after bariatric surgery? (Quantitative)
2. What are the effects of postoperative changes on patients’ quality of life? (Qualitative)
3. How do the quantitative and qualitative findings jointly contribute to the understanding of quality of life after bariatric surgery? (Mixed methods)

## Materials and methods

### Study design

A concurrent parallel mixed-method approach was used in this study [19]. Data collection and analysis processes for the quantitative (QUAN) and qualitative (QUAL) phases were conducted simultaneously. The aim of mixed-method research is to integrate different approaches in order to obtain more in-depth answers by overcoming the limitations of a single method or methodological perspective [20].

Six researchers participated in this study. The research team consisted of two psychiatric nurses (SKG and GD), two surgical nurses (NSB and DA), and two surgeons (AY and KK). While two researchers (AY and KK) had experience in bariatric surgery, the other researchers were not involved in clinical practice and had no relationship with the patients included in the study.

### Quantitative component: QUAN phase

#### Sample and eligibility criteria

The quantitative sample of this study consisted of patients who had undergone bariatric surgery at a hospital within the previous year, retrospectively identified from June 2, 2022 (June 2, 2021 – June 2, 2022). As the aim of the study was to examine the early effects following bariatric surgery, the sample was deliberately limited to the first 12 months after surgery. The early postoperative period is considered a phase during which physical recovery, psychological adjustment, and changes in quality of life are more dynamic [21, 22].

Patients who had undergone bariatric surgery within the specified time frame were identified through retrospective screening of hospital records by the responsible physicians (AY and KK). During this screening process, a total of 99 patients who met the inclusion and exclusion criteria were identified. Of those who initially agreed to participate, four later declined participation, and the quantitative phase was completed with 95 patients.

The inclusion criteria were being between 18 and 65 years of age and being at the 1st, 3rd, 6th, or 12th month follow-up after bariatric surgery. Follow-up visits were conducted during the corresponding follow-up month. Deviations of up to ± 10 days from the scheduled follow-up date were accepted to accommodate routine clinical scheduling. The exclusion criteria were defined as the presence of an active psychiatric diagnosis, chronic medical conditions that could significantly affect quality of life, and cognitive or sensory impairments that could interfere with communication. Since psychiatric diagnoses have been reported to have strong and independent effects on quality of life, psychological adjustment, and postoperative outcomes following bariatric surgery [23], and given that the heterogeneous impact of chronic medical conditions on quality of life may create significant confounding effects [24], these criteria were deliberately determined as methodological choices in sample selection.

### Data collection process

The quantitative data collection process was conducted between June 2022 and November 2022, during patients’ routine outpatient follow-up visits. Post-bariatric surgery follow-up appointments were carried out by physicians at the outpatient clinic of the relevant hospital every Wednesday between 09:00 and 12:30.

At our center, patients who undergo bariatric surgery are followed according to a standard postoperative care and monitoring protocol. Oral intake is initiated on postoperative day 2, and clinically stable patients are generally discharged on day 4. After discharge, patients attend a follow-up visit in the first week and are subsequently scheduled for routine outpatient follow-ups at the 1st, 3rd, 6th, and 12th months; after the first year, follow-ups continue annually. The first 12 months after bariatric surgery constitute a critical adjustment period during which weight loss is most pronounced and patients’ ability to maintain lifestyle changes becomes evident [25, 26]. Therefore, the effects of relatively stable personality patterns and emotion regulation difficulties [27, 28] on early postoperative quality of life were evaluated.

The distribution of participants according to postoperative follow-up months was determined by the natural flow of outpatient admissions. The relatively lower number of participants at the 12th-month follow-up was associated with a decrease in the number of patients attending routine control visits during this period and the limited number of patients accessible to the researchers. This imbalance among follow-up months was considered within the context of the study’s sampling-based structure and real-world clinical follow-up conditions.

The routine outpatient appointment dates of patients who met the eligibility criteria and agreed to participate were shared by the responsible physicians (AY and KK) with the researchers responsible for quantitative data collection (SKG and NSB). Accordingly, the cross-sectional questionnaire was administered during previously scheduled outpatient visits to patients who were within the first year after bariatric surgery (at the 1st, 3rd, 6th, or 12th month follow-up). As patient follow-up at the center is carried out according to a standardized monitoring protocol, these follow-up intervals correspond to the routine clinical assessment schedule.

The questionnaire was administered on a voluntary basis. Prior to data collection, participants were provided with detailed information about the study, and written informed consent was obtained from all participants.

### Data collection instruments

#### Personal information form

The form developed by the researchers includes variables such as age, gender, marital status, education level, type of surgery, and postoperative follow-up time.

### Inventory of Personality Organization (IPO-TR)

Developed by Kernberg and Clarkin (1995), the IPO is a 31-item self-report scale designed to assess the level of an individual’s personality organization [29]. Items are rated on a 5-point Likert scale (1 = Never true, 5 = Always true). The Turkish adaptation (IPO-TR) was conducted by Ceran Yıldırım and Yüksel (2021). The scale consists of three subdimensions: Primitive Defenses (PD), Identity Diffusion (ID), and Reality Testing (RT). Low scores on PD, ID, and RT indicate neurotic personality organization; high PD and ID with relatively low RT indicate borderline personality organization; and markedly high RT indicates psychotic personality organization. However, the IPO-TR is a self-report instrument designed to assess personality functioning and is not intended to be used as a standalone measure to determine levels of personality organization or to establish clinical diagnoses. In the original version, Cronbach’s alpha coefficients were reported as 0.81 for PD, 0.88 for ID, and 0.88 for RT. In the Turkish adaptation study, these values were 0.77, 0.82, and 0.85, respectively [30]. In the present study, the Cronbach’s alpha coefficient was found to be 0.87.

### Difficulties in Emotion Regulation Scale – short form (DERS-16)

In this study, the 16-item DERS-16 developed by Bjureberg et al. (2016) and adapted into Turkish by Yiğit and Güzey-Yiğit (2017) was used [31, 32]. The scale consists of the subdimensions of clarity, goals, impulse, strategies, and non-acceptance. It is rated on a 5-point Likert scale (1 = Almost never, 5 = Almost always). Total scores range from 16 to 80, with higher scores indicating greater difficulty in emotion regulation. In the Turkish adaptation study, the total Cronbach’s alpha coefficient was reported as 0.92 [32]. In the present study, the Cronbach’s alpha coefficient was calculated as 0.88.

### Postbariatric Surgery Quality of Life Scale (PBSQLS)

The scale consists of two main dimensions and a total of 25 items. The first dimension, Biopsychosocial Area, includes 18 items and comprises three sub-dimensions: Psychosocial Area, Physical Function, and Sexual Life. The second dimension, Complications, consists of seven items. The scale is a 5-point Likert-type measure with response options ranging from “Absolutely agree” to “Absolutely disagree.” Scores for the Biopsychosocial Area dimension range from 18 to 90, with higher scores indicating better quality of life following bariatric surgery. The Physical Function sub-dimension is reverse scored. Scores for the Complications dimension range from 7 to 35, with lower scores indicating fewer complications experienced by the patient. The Complications subscale assesses participants’ self-reported perceptions of postoperative complications and symptoms rather than objectively verified clinical complications. The reported Cronbach’s alpha coefficients are 0.884 for the Biopsychosocial Area dimension and 0.702 for the Complications dimension [33]. In this study, the Cronbach’s alpha coefficient of the scale was calculated as 0.82.

### Data analysis

Quantitative data were analyzed using SPSS Statistics version 25.0, and statistical significance was set at *p* < .05. Descriptive statistics were used to summarize participant characteristics, and all assumptions for linear regression analyses were verified before analysis [34–37]. Variables significantly associated with the outcome variables in the preliminary analyses were included as covariates in the regression models. Hierarchical multiple linear regression was used for the PBSQLS–Complications subscale and multiple linear regression for the PBSQLS–Biopsychosocial Area subscale. Effect sizes were reported using standardized regression coefficients (β), explained variance (R²), and change in explained variance (ΔR^2^) [34, 38].

### Qualitative component: QUAL phase

#### Participants and sampling strategies

The qualitative data collection process was conducted concurrently with the quantitative phase between June 2022 and November 2022. In total, 18 participants were interviewed in the qualitative phase. Participant characteristics are presented in Table 1.

**Table 1:** Characteristics of the participants who participated in the semi-structured interviews (*n*=18)

Participant number	Age(years)	Gender	Marital status	Education	Post-operative follow-up month	Type of Surgery
1	24	Female	Single	High school graduate	1st month	Obesity, sleeve
2	31	Female	Married	High school graduate	3rd months	Obesity, sleeve
3	28	Male	Single	Undergraduate degree	12th months	Obesity, sleeve
4	27	Female	Married	Undergraduate degree	6th months	Obesity, sleeve
5	28	Male	Single	Undergraduate degree	12th months	Obesity, sleeve
6	35	Male	Married	Primary education graduate	1st month	Obesity, sleeve
7	37	Female	Married	High school graduate	6th months	Obesity, sleeve
8	36	Female	Married	Primary education graduate	1st month	Obesity, bypass
9	32	Male	Single	High school graduate	6th months	Obesity, sleeve
10	29	Male	Single	Undergraduate degree	3rd months	Obesity, sleeve
11	33	Male	Married	High school graduate	1st month	Obesity, sleeve
12	35	Female	Single	High school graduate	3rd months	Obesity, sleeve
13	42	Female	Married	Primary education graduate	6th months	Obesity, sleeve
14	44	Male	Married	Primary education graduate	6th months	Obesity, bypass
15	26	Male	Married	High school graduate	3rd months	Obesity, sleeve
16	38	Female	Married	Primary education graduate	3rd months	Obesity, sleeve
17	24	Male	Single	Undergraduate degree	12th months	Obesity, sleeve
18	38	Female	Single	High school graduate	12th months	Obesity, sleeve

Participants were selected from among the patients included in the quantitative phase. After completing the quantitative questionnaire during routine outpatient control visits (1st, 3rd, 6th, and 12th postoperative follow-up months), patients were informed about the qualitative phase of the study and asked whether they were willing to participate.

Purposive sampling was used, ensuring variation to represent postoperative follow-up times (1st, 3rd, 6th, and 12th months) and gender distribution. IPO-TR and DERS-16 scores were not used in selecting qualitative participants, as data collection processes were conducted simultaneously.

A total of 26 patients were invited to participate in qualitative interviews; 8 declined participation, and 18 agreed to be interviewed. Of those who declined, four were in the 1st-month follow-up period and reported that they did not wish to participate due to postoperative pain and fatigue; two were unable to participate due to work commitments, and two declined because of childcare responsibilities. Written informed consent was obtained from all participants prior to the interviews.

### Data collection

Semi-structured individual interviews were used as the data collection method. The semi-structured interview form was developed by the researchers based on the literature regarding postoperative management and psychosocial processes following bariatric surgery [6, 7, 12, 13, 39–42]. The interview guide was developed in consultation with an experienced bariatric surgeon, a psychiatrist, and a psychiatric nurse, and finalized in accordance with their feedback.

Interviews were conducted face-to-face in a meeting room independent of the hospital’s clinical areas, immediately after the same outpatient visit during which quantitative data collection had been completed.

All interviews were conducted by two researchers (SKG and NSB) who were not involved in the participants’ postoperative follow-up or care processes and who had experience in conducting in-depth interviews. It was clearly stated before the interviews that participation or non-participation would have no effect on the participants’ medical care.

Interviews were conducted in a single session; durations ranged from 19 to 50 min, with an average of approximately 35 min. Interviews were audio-recorded only. Audio recordings were securely stored by NSB, transcribed verbatim, and anonymized using codes (P1, P2, P3, etc.).

After each interview, the thematic analysis process was conducted concurrently. As interviews progressed, recurring patterns in the data were observed, and no new themes or codes emerged. Data saturation was considered to have been reached by the sixteenth interview; to confirm this, two additional interviews (17th and 18th) were conducted. No new themes or information were identified in these interviews; instead, existing themes were further reinforced. Accordingly, data collection was concluded after the 18th interview, and all interviews were included in the analysis [43].

### Data analysis

In the analysis of qualitative data, the six-phase inductive thematic analysis approach proposed by Braun and Clarke was used [44]. Audio recordings were transcribed verbatim by the second author (NSB) concurrently with the interviews, the analysis process was conducted simultaneously. Data were analyzed using the MAXQDA qualitative data analysis software.

The analysis process was carried out independently by two researchers (SKG and NSB). Both researchers reviewed all transcripts separately, identified meaningful statements, and generated initial open codes using a data-driven (inductive) approach while maintaining close adherence to participants’ expressions.

Coding discrepancies were resolved though discussion and consensus between the researchers. Similar codes were grouped into themes, which were reviewed and refined with the research team (GD and DA) to ensure coherence and conceptual integrity before finalization.

After preliminary themes were developed, transcripts were shared with participants as part of a member-checking process to ensure the accuracy of content and interpretations. Of the 18 participants, 13 provided feedback; none reported corrections, additions, or objections regarding the content or interpretations. Themes were reviewed once more in light of participant feedback and finalized.

Throughout the interviews, the semi-structured question framework was maintained, and probing questions were used only to deepen responses based on participants’ answers.

### Trustworthiness of the study

The trustworthiness of the qualitative study was evaluated according to the criteria proposed by Lincoln and Guba (1985): credibility, transferability, dependability, and confirmability [45]. The methods applied within this framework are presented in detail in Supplementary File 1.

### Integration of quantitative and qualitative components

This study was conducted using a concurrent parallel mixed-method design, in which quantitative and qualitative data collection processes were carried out within the same time frame. However, the analytical backbone of the study was formed by the quantitative data, while qualitative data were used to explain and deepen how quantitative findings were experienced within clinical and psychosocial contexts. Therefore, relatively greater emphasis was placed on quantitative findings during the analysis process (QUAN + qual) [46].

In the quantitative phase, the relationships between personality functioning, emotion regulation difficulties, and quality of life following bariatric surgery were examined using statistical analyses. The qualitative phase was designed to explore how the relationships identified—or not identified—in quantitative analyses gained meaning within individuals’ lived experiences. In this context, qualitative data were used to make visible the context and potential explanations underlying the quantitative findings.

Quantitative and qualitative data were analyzed separately. The integration process was conducted during the interpretation phase, after the completion of analyses for both data types.

During integration, quantitative analysis results (both statistically significant and non-significant findings) were first listed. Qualitative themes were then matched with these findings according to their level of conceptual overlap. This matching process was carried out by two researchers (SKG and NSB) and subsequently discussed in research team meetings (SKG, NSB, GD, and DA) until consensus was reached. The integration of findings was visualized through a joint display table presenting quantitative and qualitative results side by side. Joint displays are among the key tools in mixed-method research that enhance transparency and traceability of integration [47].

Overlaps between quantitative and qualitative findings were evaluated as convergence, and meta-inferences were generated in such cases. Findings that were not statistically significant in the quantitative analyses but emerged clearly in the qualitative data were addressed within the framework of complementarity, reflecting the nature of different data types; such findings were interpreted as explanatory results indicating potential limitations of quantitative measurements. In cases where clear divergences were observed between quantitative and qualitative data, no meta-inferences were produced, and findings were discussed within the context of methodological limitations, in line with recommendations from the mixed-method literature [48].

**Fig. 1:**
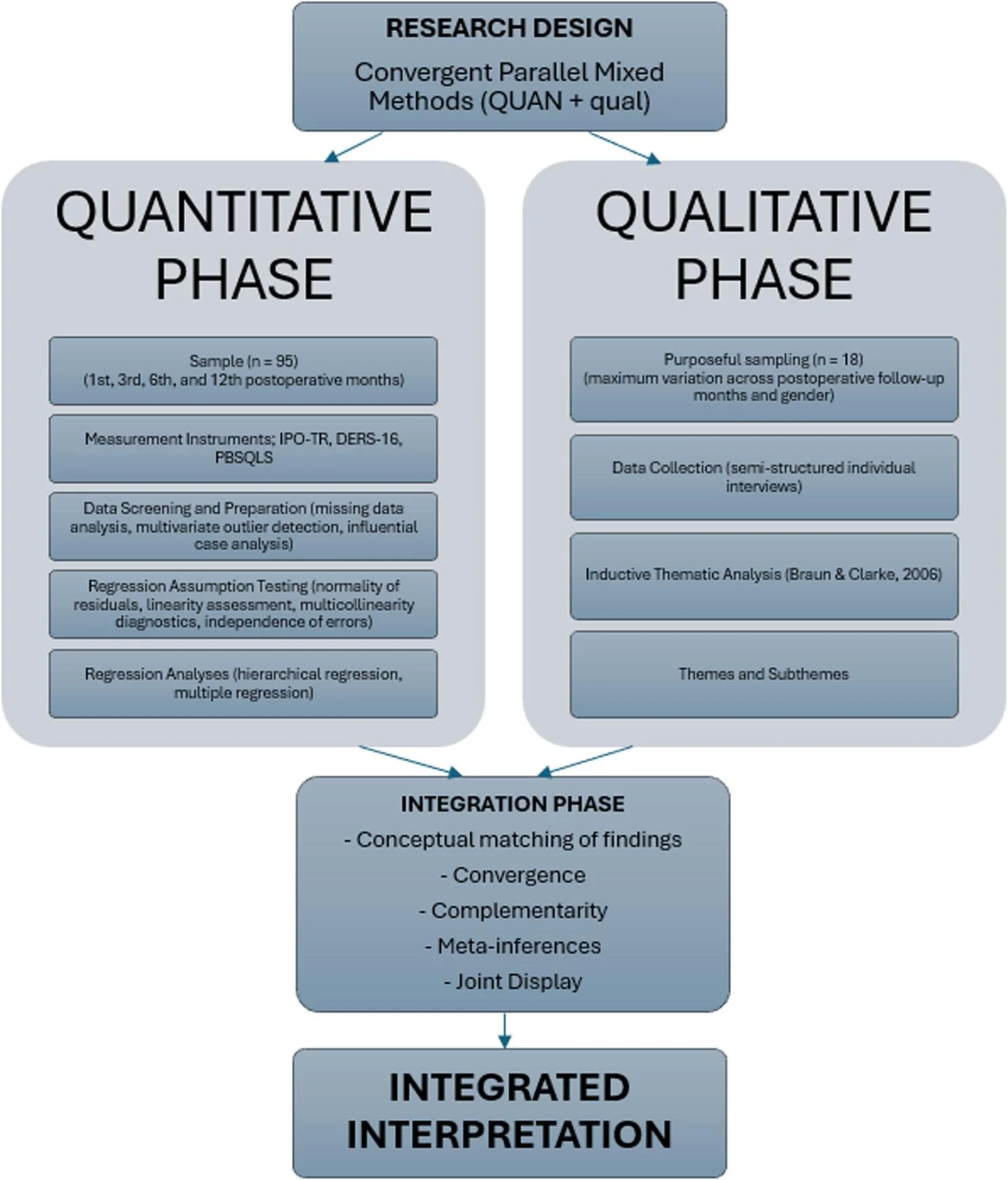
Mixed method flowchart

Meta-inferences were generated only when quantitative findings were supported or meaningfully deepened by qualitative data. This approach is consistent with methodological recommendations emphasizing that meta-inferences in mixed-method research should be produced with consideration of the epistemological boundaries of different data types [48] (Fig. 1).

### Ethical considerations

The study was approved by the Sakarya University Faculty of Medicine Non-Interventional Research Ethics Committee with the study code E-71522473-050.01.04-128294-108. Written informed consent confirming voluntary participation was obtained from all participants prior to both the quantitative and qualitative data collection phases.

During the qualitative phase, participants provided both verbal and written consent for audio recording of the interviews. Anonymity was ensured by assigning pseudonyms to participants during transcription. Interview transcripts were securely stored in password-protected environments.

If significant psychological distress, intense anxiety, hopelessness, or similar negative expressions emerged during interviews, the researchers conducting the interviews evaluated the situation clinically. With the participant’s explicit consent, the responsible physicians (AY and KK) were informed. When deemed necessary, the responsible physicians referred participants for psychiatric consultation, evaluation, and follow-up.

## Results

The findings are presented under three headings: (1) quantitative findings, (2) qualitative findings, and (3) mixed-method findings.

### Quantitative findings

#### Descriptive statistics

The total sample consisted of 95 patients with a mean age of 32.12 years (SD = 6.60). The majority of participants were female (*n* = 73, 76.8%), while 22 (23.2%) were male. Regarding marital status, 58 patients (61.1%) were married and 37 (38.9%) were single. In terms of educational level, 34 patients (35.8%) had primary education, 43 (45.3%) had completed high school, and 18 (18.9%) held a bachelor’s degree. Postoperative follow-up time varied among participants: 34 patients (35.8%) were at the 1st month, 26 (27.4%) at the 3rd month, 24 (25.3%) at the 6th month, and 11 (11.6%) at the 12th month follow-up. Regarding surgical type, the majority of patients (*n* = 87, 91.6%) underwent sleeve gastrectomy due to obesity, while 8 patients (8.4%) underwent bypass surgery.

Descriptive statistics for the study scales are presented in Table 2. The mean IPO-TR total score was 57.23 ± 20.56 (min: 32 – max: 120), and the mean DERS-16 total score was 33.66 ± 15.39 (min: 16 – max: 71). The mean PBSQLS–Complications subscale score was 20.42 ± 4.89 (min: 7 – max: 29), and the mean PBSQLS–Biopsychosocial Area subscale score was 69.61 ± 13.53 (min: 18 – max: 86) (Table 2).

**Table 2:** Descriptive statistics for study variables (*n* = 95)

Variable	Mean ± SD	Min–Max	Skewness	Kurtosis
IPO-TR Total Score	57.23±20.56	32-120	1.03	0.55
DERS-16 Total Score	33.66±15.39	16-71	0.73	-0.86
PBSQLS-Complications Subscale	20.42±4.89	7-29	-0.21	-0.50
PBSQLS-Biopsychosocial Domain Subscale	69.61±13.53	18-86	1.31	1.12

### Effects of descriptive variables

The relationships between descriptive variables (age, gender, marital status, education level, postoperative follow-up time, and type of surgery) and the PBSQLS–Biopsychosocial Area and PBSQLS–Complications subscales were examined separately using simple linear regression analyses.

Results indicated that female gender positively and significantly predicted the PBSQLS–Complications subscale total score (β = 0.21, t(93) = 2.07, *p* < .05).

The postoperative follow-up time variable was included in the model with the twelfth month as the reference category. Accordingly, patients at the first-month follow-up had significantly higher PBSQLS–Complications subscale total scores compared to those at the twelfth month (β = 0.74, t(91) = 4.99, *p* < .001). Similarly, patients at the third month (β = 0.53, t(91) = 3.72, *p* < .001) and sixth month (β = 0.31, t(91) = 2.04, *p* < .05) follow-ups also had significantly higher PBSQLS–Complications subscale total scores compared to those at the twelfth month. No other descriptive variables had a statistically significant effect on the PBSQLS–Complications subscale total score (all *p* > .05).

The effects of descriptive variables on the PBSQLS–Biopsychosocial Area subscale total score were also examined using simple linear regression analyses; however, none of these variables significantly predicted the PBSQLS–Biopsychosocial Area subscale total score (all *p* > .05).

Gender and postoperative follow-up month, which showed significant effects on the PBSQLS–Complications subscale total score, were included as covariates in subsequent analyses.

### Hierarchical regression results (PBSQLS–Complications subscale)

Hierarchical multiple linear regression analysis was conducted to examine the relationship between IPO-TR, DERS-16, and the PBSQLS–Complications subscale total score. In the first model, gender (male as the reference category) and dummy variables representing postoperative follow-up times (1st, 3rd, and 6th months, with the 12th month as the reference category) were included as covariates. In the second model, IPO-TR and DERS-16 total scores were added to these variables.

The first model, which included the covariates, was statistically significant (F(4,90) = 7.78, *p* < .001; R² = 0.26; Adj. R² = 0.22). With the addition of IPO-TR and DERS-16 in the second model, the proportion of explained variance increased significantly, and the overall model was found to be statistically significant (F(6,88) = 12.47, *p* < .001; R² = 0.46; Adj. R² = 0.42). The increase in explained variance between the models was statistically significant, indicating a 20.0% increase in explained variance with the inclusion of IPO-TR and DERS-16 (ΔR² = 0.20).

In the second model, IPO-TR scores positively and significantly predicted the PBSQLS–Complications subscale total score (B = 0.16; SE = 0.03; β = 0.62; t(88) = 4.57; *p* < .001; 95% CI [0.09; 0.23]). In contrast, after IPO-TR was included in the model, the predictive effect of DERS-16 scores on the PBSQLS–Complications subscale total score was not statistically significant (B = − 0.08; SE = 0.05; β = −0.24; t(88) = − 1.72; *p* = .089; 95% CI [− 0.17; 0.01]) (Table 3).

**Table 3:** Hierarchical multiple linear regression analysis results for the prediction of PBSQLS–Complications subscale total score by IPO-TR and DERS-16

		B	SE	β	t	%95 GA	F	R	R^2^	R^2^_adj_	ΔR²
Model 1	(Constant)	13.42	1.52		8.84**		7.78**	0.51	0.26	0.22	
	Female	1.38	1.14	0.11	1.21	[−0.89; 3.65]					
	1st month	7.54	1.63	0.70	4.62**	[4.30; 10.78]					
	3rd month	5.85	1.67	0.50	3.50**	[2.53; 9.18]					
	6th month	3.45	1.69	0.29	2.04*	[0.10; 6.81]					0.20
Model 2	(Constant)	6.97	1.74		4.00**		12.47**	0.68	0.46	0.42	
	Female	1.69	0.99	0.11	1.41	[−0.57; 3.36]					
	1st month	7.36	1.41	0.68	5.22**	[4.56; 10.16]					
	3rd month	6.08	1.44	0.52	4.21**	[3.21; 8.95]					
	6th month	3.77	1.46	0.32	2.59*	[0.88; 6.67]					
	IPO-TR	0.16	0.03	0.62	4.57**	[0.09; 0.23]					
	DERS-16	-0.08	0.05	-0.24	-1.72	[−0.17; 0.01]					

*B* unstandardized regression coefficient, *SE* standard error, *β* standardized regression coefficient, *CI* confidence interval

Model 1 includes gender (0 = male, 1 = female) and postoperative follow-up time (reference category = 12th month); Model 2 additionally includes IPO-TR and DERS-16 scores

ΔR² represents change in explained variance between models

*p < .05, **p < .001

### Multiple regression results (PBSQLS–Biopsychosocial Area subscale)

Multiple linear regression analysis was conducted to examine the effects of IPO-TR and DERS-16 on the PBSQLS–Biopsychosocial Area subscale (total score including psychosocial area, physical function, and sexual life subdimensions). The overall model was statistically significant (F(2,92) = 3.36, *p* < .05).

IPO-TR scores negatively and significantly predicted the PBSQLS–Biopsychosocial Area subscale total score (B = − 0.39; SE = 0.15; β = −0.45; t(92) = − 2.58; *p* = .011; 95% CI [− 0.69; −0.09]). In contrast, the predictive effect of DERS-16 scores on the PBSQLS–Biopsychosocial Area subscale total score was not statistically significant, although it was at a borderline level (*p* = .050) (Table 4).

**Table 4:** Multiple linear regression analysis results for the prediction of PBSQLS–Biopsychosocial Area subscale by IPO-TR and DERS-16

Dependent Variable	Independent Variable	B	SE	β	t	%95 GA	F	R	R^2^	R^2^_adj_
Biopsychosocial domain	Constant	28.72	5.26		5.46**		3.36*	0.26	0.07	0.05
	*IPO-TR*	-0.39	0.15	-0.45	-2.58*	[−0.69; −0.09]				
	*DERS-16*	0.40	0.20	0.35	1.99	[0.00; 0.80]				

*B* unstandardized regression coefficient, *SE* standard error, *β* standardized regression coefficient, *CI* confidence interval

Model includes IPO-TR and DERS-16 as independent variables

*p < .05, **p < .001

### Qualitative findings

Eighteen patients who attended postoperative follow-up visits after bariatric surgery participated in the semi-structured interviews. The characteristics of these participants are presented in Table 1.

Analysis of the qualitative interview recordings resulted in the identification of four main themes: (1) psychological well-being, (2) daily life and functional capacity, (3) interpersonal relationships, and (4) personality traits and adaptation dynamics (Table 5).

**Table 5:** Themes and subthemes related to post-bariatric surgery experiences

Themes	Subthemes
Psychological Well-Being	Psychological distress at the first and third postoperative months
	Body image and self-confidence at the sixth and twelfth postoperative months
	Fear of weight regain
Daily Life and Functional Capacity	Improvement in sleep quality and physical pain
	Psychological burden of dietary changes
	Increased physical activity and social participation
Interpersonal Relationships	Preoperative lack of support and fear
	Postoperative support and motivation
	Jealousy and relational tension
Personality Traits and Adaptation Dynamics	Externalization of responsibility
	Self-Criticism and perceived inadequacy
	Skepticism and uncertainty

### Theme 1: Psychological well-being

This theme encompasses participants’ emotional experiences accompanying the surgical process and their psychological transformation over time. Narratives indicate that the sense of vulnerability and dependency, which was more prominent during the 1st–3rd postoperative months, was replaced by body satisfaction and increased self-confidence during the 6th–12th postoperative months. However, persistent fear of weight regain emerged as a continuous dimension of this theme.

#### Psychological distress at the first and third postoperative months

Accounts from participants in the first and third month follow-ups centered on physical dependency and the need for self-care. Inability to perform daily activities due to pain and limited mobility led to feelings of regret, anger, and inadequacy. This period stood out as a phase marked by ambivalence toward the surgery.

*“My husband went to work during the day and looked after me and my daughter in the evening. I felt that he was very tired. At that time I was very angry with myself*,* ‘Why did I have the operation?’ I really regretted it. I really regretted it. I couldn’t even look after my daughter all day.” [31 years old*,* female*,* 3 months postoperative]*.

#### Body image and self-confidence at the sixth and twelfth postoperative months

At the sixth and twelfth month follow-ups, participants expressed satisfaction with their bodies, reduced feelings of shame, and increased self-confidence. This transformation in body image was not limited to physical appearance; it also had positive effects on social relationships and future plans.

*“I’m someone who loves wearing colorful clothes. During that time*,* I couldn’t find anything I wanted for myself. Now I’ve gone from size 6X to XL. I constantly go out and shop for myself. This has affected me positively on a psychological level.” [42 years old*,* female*,* 6 months postoperative]*.

*“When I was overweight I was embarrassed*,* shy. It was good for me to be in the dark. I am now more tolerant of my girlfriend seeing me naked.” [28 years old*,* male*,* 12 months postoperative]*.

#### Fear of weight regain

A pronounced fear of weight regain accompanied weight loss. Participants reported remaining constantly vigilant about their eating behaviors, and even minor deviations from their routines generated anxiety.

*“I can’t finish all my food because I’m scared—what if I gain weight again? I’m worried about going back to square one.” [35 years old*,* female*,* 3 months postoperative]*.

*“Because my stomach has shrunk*,* I can’t eat much. But if I eat even a little more than usual*,* thoughts about returning to my previous weight don’t leave me alone.” [38 years old*,* female*,* 12 months postoperative]*.

### Theme 2: Daily life and functional capacity

This theme encompasses the effects of bariatric surgery on individuals’ physical functioning and daily life practices. Participants reported significant improvements in sleep quality and mobility; however, they stated that dietary changes created a mental burden, particularly during the 1st–3rd postoperative months. Physical recovery was observed to increase social participation.

#### Improvement in sleep quality and physical pain

Participants reported improved sleep quality and reductions in pain and respiratory problems during the postoperative period. This improvement was associated with decreased daytime fatigue and an enhanced sense of overall well-being.

*“At the end of the day*,* my back and knees were very painful. When I went to bed*,* I couldn’t sleep because of the pain. I often woke up with a feeling of suffocation; I was afraid of dying in my sleep. Now I can sleep on my back*,* and the pain is not enough to keep me awake.” [44 years old*,* male*,* 6 months postoperative]*.

#### Psychological burden of dietary changes

Strict dietary rules, particularly during the first three months, created a mental burden and a sense of social isolation among participants. Feelings of guilt and regret were also reported during the process of adapting to the diet.

*“The first 2 months were very difficult; I was tired of eating soup. My family did not eat with me because I was sad when I saw them. I remember that I regretted having surgery at that time*,* and I cried a lot.” [27 years old*,* female*,* 6 months postoperative]*.

#### Increased physical activity and social participation

In the later months, participants reported increased physical mobility and more active participation in work and social life. A reduction in social isolation was associated with a decrease in depressive feelings.

*“I used to get out of breath while walking. Walking or standing for long periods physically exhausted me. At that time*,* I couldn’t accompany my friends*,* so I would say I’d rather stay at home. I often found myself crying helplessly. But now it’s the opposite. I hardly stay at home anymore. I even started a new job. Being where I want without shame or difficulty—I’m just grateful*,* I’m happy…” [28 years old*,* male*,* 12 months postoperative]*.

*“It wasn’t possible for me to attend any activity; I would get tired very quickly. Now a concert or a camping trip is no longer torture for me.” [32 years old*,* male*,* 6 months postoperative]*.

### Theme 3: Interpersonal relationships

This theme encompasses the effects of the surgical process on family and partner relationships. While lack of support and fear-based narratives were prominent in the preoperative period, supportive attitudes and increased motivation became more evident after surgery. However, weight loss was also observed to generate jealousy and tension in some relationships.

#### Preoperative lack of support and fear

Family and partner resistance emerged as a prominent theme in the preoperative period. Discourses associating surgery with death created feelings of loneliness and being misunderstood among participants.

*“My family was very against the surgery. They were telling me what they heard about people who died from the operation. They did not want it at all; they thought I would die. I was very lonely.” [29 years old*,* male*,* 3 months postoperative]*.

#### Postoperative support and motivation

After surgery, participants reported that their spouses and families actively accompanied them in lifestyle changes. This support was described as a motivating factor.

*“My wife both prepares my meals and avoids eating anything different from what I eat. Even if I say*,* ‘Don’t worry about me*,* eat whatever you want*,*’ she doesn’t listen. She also joins me on my walks. If I forget or get tired*,* she suggests going for a walk. Her behavior motivates me.” [35 years old*,* male*,* 1 month postoperative]*.

#### Jealousy and relational tension

Participants stated that after weight loss, jealousy and controlling behaviors emerged in their partners.

*“My husband was very supportive at the beginning. As I started to lose weight*,* I began dressing more colorfully and attractively. I now have a social life and receive compliments from people. But I feel that my husband is uncomfortable with this. He tries to restrict me and blames me*,* saying that I have changed.” [37 years old*,* female*,* 6 months postoperative]*.

*“After my body became smaller*,* I started buying colorful*,* tighter clothes. They suit me very well—I can see it in the mirror. But each time*,* my husband says they don’t suit me and asks me to wear loose clothing.” [42 years old*,* female*,* 6 months postoperative]*.

### Theme 4: Personality traits and adaptation dynamics

This theme encompasses how participants evaluated their postoperative adjustment process and the cognitive patterns that emerged during this period. Findings indicated that while some participants attributed their adjustment difficulties to external factors, others evaluated themselves in a self-critical manner. Additionally, skepticism toward healthcare professionals and concerns about social perceptions emerged as significant dynamics accompanying the process.

#### Externalization of responsibility

Participants associated difficulties in adhering to treatment with environmental factors. Negative comments about the surgery were described as undermining motivation.

*“Before I had the surgery*,* I was getting a lot of bad comments about my weight. I was upset*,* so I had the surgery*,* and this time they said*,* ‘Why did you have the surgery? You could have lost this weight by eating less*,*’ or ‘Did you risk dying because of your weight?’ These comments do nothing but drag me down.” [38 years old*,* female*,* 3 months postoperative]*.

*“I couldn’t keep up with the exercise—bad luck. I’m supposed to walk regularly*,* but the weather is cold. Even when the weather is good*,* something always comes up.” [26 years old*,* male*,* 3 months postoperative]*.

#### Self-criticism and perceived inadequacy

Participants reported anxiety about whether they were adhering sufficiently to treatment recommendations and evaluated themselves critically.

*“My doctor told me what I can and cannot eat. But I still don’t know what to do; I’m undecided. Am I doing everything well enough?” [36 years old*,* female*,* 1 month postoperative]*.

*“When I compare myself to other patients*,* I don’t think I’ve lost that much weight. Even if I follow what the doctor says*,* maybe more was needed. I couldn’t manage the process properly.” [27 years old*,* female*,* 6 months postoperative]*.

#### Skepticism and uncertainty

Participants expressed experiencing internal doubt during the postoperative process despite reassurances from healthcare professionals, and stated that lack of detailed information caused discomfort. This skepticism also extended to social perceptions following bodily changes. Some participants reported feeling that others were evaluating them negatively.

*“They (doctors) say*,* ‘You are doing very well; there is nothing wrong with you.’ But I feel like there’s something wrong and it’s being glossed over. It bothers me not knowing the details.” [33 years old*,* male*,* 1 month postoperative]*.

*“After losing a lot of weight*,* there are involuntary sagging and changes in the way you walk. I can sense people’s looks; I feel like they think negatively about me.” [38 years old*,* female*,* 12 months postoperative]*.

### Mixed-methods findings (integration)

Findings were evaluated integratively after the quantitative and qualitative analyses were completed separately. During the integration process, statistically significant and non-significant quantitative results were systematically listed and matched with qualitative themes according to their level of conceptual overlap. Overlapping patterns were interpreted as convergence, whereas findings that were not statistically significant in the quantitative phase but became evident in the qualitative data were interpreted within the framework of complementarity. The joint display is presented in Table 6.

**Table 6:** Joint presentation of quantitative and qualitative findings

Quantitative Findings	Qualitative Theme	Nature of Integration	Integrated Interpretation
IPO-TR significantly and positively predicted the PBSQLS–Complications subscale (β = 0.62; p < .001).	Theme 4 – Personality traits and adaptation dynamics (Subthemes: Skepticism and uncertainty; Self-criticism and perceived inadequacy)	Convergence	As the level of impairment in personality patterns increased, complication scores also increased. Qualitative findings suggest that participants with higher IPO-TR scores more frequently described experiences characterized by mistrust, uncertainty, and self-critical evaluations. These qualitative themes provide contextual insight into participants’ self-reported postoperative experiences but do not indicate objectively verified postoperative complications.
When IPO-TR was included in the model, DERS-16 was not a significant predictor of the PBSQLS–Complications subscale (p = .089).	Theme 1 – Psychological well-being (Subtheme: Psychological distress at the first and third postoperative months)	Complementarity	Although emotional distress was reported in the qualitative data, trait-level emotion regulation difficulties did not independently predict complication scores. This suggests that emotional reactions may fluctuate in a context-specific manner.
IPO-TR negatively predicted the PBSQLS–Biopsychosocial Area subscale (β = −0.45; p = .011).	Theme 1 – Psychological well-being; Theme 3 – Interpersonal relationships	Convergence	As the level of impairment in personality patterns increased, biopsychosocial quality of life scores were lower. Qualitative themes indicate that fragile self-structure, relational tension, and cognitive sensitivity may be associated with perceptions of quality of life.
The 1st, 3rd, and 6th postoperative month groups reported higher PBSQLS–Complications scores compared to the 12th month.	Theme 1 – Psychological well-being (Subtheme: Psychological distress at the first and third postoperative months); Theme 2 – Daily life and functional capacity (Subtheme: Increased physical activity and social participation)	Convergence	Higher complication scores were reported in earlier follow-up periods compared to the 12th month. Qualitative data indicate that psychological vulnerability and adjustment difficulties were more pronounced during these periods. Improvements in body image and social functioning reported in later months were observed alongside lower complication scores.
No significant demographic predictors were found for the PBSQLS–Biopsychosocial Area subscale.	Theme 3 – Interpersonal relationships; Theme 4 – Personality traits and adaptation dynamics (Subtheme: Externalization of responsibility)	Complementarity	Demographic variables did not statistically predict biopsychosocial scores; however, qualitative findings indicate that relational and contextual processes are associated with the experience of quality of life.

### Integration related to the PBSQLS–complications subscale

Quantitative analyses indicated that IPO-TR scores strongly and positively predicted the PBSQLS–Complications subscale. Qualitative findings make the experiential context of this quantitative relationship visible. Narratives characterized by skepticism, uncertainty, and self-critical tendencies provide contextual insights into how participants make sense of their postoperative experiences. Participants’ continued doubts about the process despite receiving medical reassurance point to the complex and multidimensional nature of postoperative experiences. Taken together, these findings indicate a statistically consistent association between personality functioning and self-reported scores on the PBSQLS–Complications subscale, while the qualitative findings provide contextual insights that help interpret this association. However, because the PBSQLS–Complications subscale reflects participants’ self-reported perceptions of postoperative complications and symptoms, these findings should not be interpreted as representing objectively verified postoperative complications.

Second, quantitative analyses showed that emotion regulation difficulties (DERS-16) did not demonstrate an independent association with PBSQLS–Complications scores. However, qualitative data suggested that intense emotional reactions (regret, anger, anxiety) were particularly prominent during the 1st–3rd postoperative months. This may be related to the quantitative measure being more sensitive to trait-level general emotion regulation difficulties, whereas qualitative narratives may better capture time- and context-specific situational emotional fluctuations.

### Integration related to the PBSQLS–Biopsychosocial area subscale

The negative association between IPO-TR scores and the PBSQLS–Biopsychosocial subscale indicates a significant relationship between personality functioning and self-reported quality of life. The qualitative findings provide contextual insights into how this relationship is experienced by patients. During the 1st–3rd postoperative months, participants reported vulnerability, ambivalence, and feelings of inadequacy; in contrast, during the 6th–12th postoperative months, body satisfaction, increased self-confidence, and strengthened social participation were observed. Additionally, within the theme of interpersonal relationships, dynamics such as support, jealousy, and relational tension were found to be associated with perceptions of quality of life. Taken together, these findings help contextualize the quantitatively identified associations by illustrating how they are reflected in patients’ subjective experiences.

### Integration related to the time variable

In the quantitative analyses, postoperative follow-up time was included in the model with the 12th month as the reference category, and it was determined that the 1st, 3rd, and 6th month groups had significantly higher PBSQLS–Complications scores compared to the 12th month. This finding indicates that higher complication scores were reported in earlier follow-up periods relative to the 12th month. Qualitative data revealed that intense psychological experiences such as physical dependency, regret, and anger were prominent during the 1st–3rd postoperative months, whereas in later periods (6th–12th months), body image and social participation strengthened. This holistic evaluation suggests that higher complication scores in earlier follow-up periods compared to the 12th month may be associated with psychological vulnerability. However, due to the cross-sectional nature of the study, no causal inference regarding change over time is made.

### Complementary findings

In quantitative analyses, some descriptive variables did not show a significant effect on the biopsychosocial domain. However, qualitative data demonstrated that experiential dimensions such as social support, externalization, and relational tension played a prominent role in perceptions of quality of life. This indicates that contextual and relational processes not captured by quantitative measures became visible through qualitative data.

Overall, when the quantitative and qualitative findings are considered together, quality of life following bariatric surgery appears to be more than merely an outcome of physical recovery. While the quantitative findings identified associations between psychological variables and quality of life, the qualitative findings provided insights into how these associations were experienced in patients’ daily lives, interpersonal relationships, and postoperative adjustment processes. The integration of these findings suggests that quality of life following bariatric surgery is a multidimensional experience that may be associated with individual, interpersonal, and contextual processes. However, given the cross-sectional design and the reliance on self-report measures, these associations should not be interpreted as indicating causal relationships.

## Discussion

In this concurrent parallel mixed-methods study, quality of life following bariatric surgery was examined from a multidimensional perspective in the context of personality functioning and emotion regulation. Quantitative analyses identified significant associations between self-reported personality functioning, as assessed by the IPO-TR, and self-reported scores on the PBSQLS–Complications and PBSQLS–Biopsychosocial subscales, whereas the qualitative findings provided contextual insights into how these associations were reflected in patients’ lived experiences. Taken together, the findings suggest that, in addition to biomedical recovery, self-reported personality functioning, cognitive appraisal processes, and interpersonal relationship dynamics may be associated with how quality of life is experienced following bariatric surgery.

### Personality functioning and self-reported postoperative complications

Quantitative findings showed that IPO-TR scores were strongly and positively associated with the PBSQLS–Complications subscale. This finding indicates a significant association between self-reported personality functioning, as assessed by the IPO-TR, and self-reported scores on the PBSQLS–Complications subscale. The findings suggest that the manner in which individuals experience and evaluate the postoperative period may be important in understanding this relationship. Consistent with this interpretation, the qualitative findings revealed that experiences such as uncertainty, skepticism, self-criticism, and difficulties in adjustment were frequently reported by some participants during the postoperative period. The fact that some participants continued to express doubts about the postoperative process despite reassurance from healthcare professionals suggests that individual differences may exist in how postoperative experiences are appraised and interpreted [49, 50]. This finding indicates that self-reported scores on the PBSQLS–Complications subscale may, at least in part, reflect participants’ subjective evaluations of their postoperative experiences. However, these findings should not be interpreted as representing objectively verified postoperative complications.

Feelings of uncertainty regarding the postoperative period increased anxiety in some participants and created difficulties in treatment adherence. The perception that “everything is fine” explanations from the healthcare team lacked sufficient detail and therefore failed to provide reassurance may be interpreted as a clinical reflection of the need for structured and clear information. These observations are consistent with the literature suggesting that personality organization characteristics, as assessed by the IPO-TR, may be associated with the ways individuals manage stressful experiences and navigate interpersonal situations [51, 52]. Therefore, scores obtained from the PBSQLS–Complications subscale may reflect participants’ self-reported postoperative experiences. These scores should not be assumed to correspond directly to objectively verified clinical complications.

These findings are consistent with previous studies reporting associations between personality functioning, as assessed by the IPO-TR, and treatment outcomes. Koelen et al. reported that baseline personality organization levels were moderately to strongly associated with better treatment outcomes [18]. Similarly, Di Mattei et al. demonstrated that primitive defense mechanisms are associated with poorer psychological adjustment [53]. Within this framework, individuals with higher IPO-TR scores may be more likely to subjectively appraise stressful medical experiences as more challenging. However, this interpretation should be considered within the context of the correlational findings derived from self-report data.

### Personality functioning and biopsychosocial quality of life

The negative association between IPO-TR scores and the PBSQLS–Biopsychosocial subscale indicates a significant relationship between IPO-TR scores and self-reported quality of life scores. This finding suggests that biopsychosocial well-being following bariatric surgery may be associated not only with improvements in physical functioning but also with the ways individuals perceive and evaluate themselves, as well as their relational experiences.

Qualitative themes support this relationship contextually. During the 1st–3rd postoperative months, vulnerability, ambivalence, and feelings of inadequacy were prominent; in later periods (6th–12th months), body satisfaction, increased self-confidence, and strengthened social participation were observed. Additionally, under the theme of interpersonal relationships, not only supportive attitudes but also jealousy and relational tensions became evident, suggesting that biopsychosocial quality of life includes a relational component. Taken together, these findings suggest that biopsychosocial quality of life may be associated with self-reported personality functioning and the interpersonal context in which patients experience the postoperative period.

### Emotion regulation difficulties

In this study, emotion regulation difficulties (DERS-16) did not demonstrate an independent and strong association with postoperative complications or quality of life in quantitative models. However, qualitative data revealed that intense emotional reactions such as regret, anger, and anxiety were particularly evident in the early postoperative period. The discrepancy between quantitative and qualitative findings should be interpreted not as a superficial contradiction but within methodological and theoretical differences related to measurement level and evaluative context.

First, DERS-16 primarily reflects trait-level emotion regulation difficulties, whereas qualitative interviews captured situational emotional reactions concentrated during specific postoperative periods. Emotional fluctuations experienced during the early postoperative period may represent temporary responses specific to contextual stressors and therefore may not have emerged as independent predictors in a scale measuring general emotion regulation capacity. This highlights the time- and context-dependent nature of emotional experiences.

Second, emerging evidence suggests that assessing emotional functioning in obesity samples solely through self-report measures may be limited [54]. During periods of intense stress, individuals’ capacity to differentiate and label emotions may diminish; thus, self-report instruments may measure cognitive representations of emotional experiences rather than the depth of the lived emotional process. Therefore, the absence of statistically significant findings for emotion regulation difficulties in quantitative models does not necessarily indicate clinical insignificance.

Additionally, socially desirable responding may have contributed to this discrepancy. During the bariatric surgery process, individuals may be inclined to present themselves as adherent and “successful” patients. This tendency may lead to underreporting of emotional difficulties in self-report measures and weaken observed relationships in quantitative models.

Within this framework, the lack of statistically significant associations for emotion regulation difficulties in quantitative models should not be interpreted as evidence of clinical irrelevance. Rather, it should be considered in light of measurement scope, evaluative context, and structural relationships among variables [55]. The mixed-method approach enabled examination beyond self-report data, making visible the dynamic nature of emotional responses shaped by temporal and environmental conditions.

### Postoperative follow-up time and the adjustment process

When postoperative follow-up time was included in quantitative models with the 12th month as the reference category, the 1st, 3rd, and 6th month groups had higher PBSQLS–Complications scores compared to the 12th month. This finding indicates that higher complication scores were reported during earlier follow-up periods.

Qualitative findings revealed that intense psychological experiences such as physical dependency, self-care difficulties, regret, and anger were prominent in the early period, whereas body image and social participation strengthened in later months. This pattern suggests that higher self-reported scores on the PBSQLS–Complications subscale during the early postoperative period may co-occur with experiences of psychological vulnerability and uncertainty. However, due to the cross-sectional design of the study, causal inferences regarding changes over time cannot be made.

### Demographic variables and psychosocial context

The study showed that female gender was associated with PBSQLS–Complications scores and that earlier postoperative follow-up periods were associated with higher complication scores. In contrast, some demographic variables did not emerge as significant predictors of the biopsychosocial domain in quantitative analyses. However, qualitative findings indicated that experiential dimensions such as social support, externalization, and relational tension played a significant role in perceived quality of life. This suggests that contextual and relational processes not captured by quantitative measures became visible through qualitative data.

The literature reports conflicting findings regarding gender differences in complication reporting. A meta-analysis by Risi et al. indicated that women were more exposed to long-term postoperative complications [56], whereas Dharap et al. found no gender differences [57]. Husain et al. identified male gender as a risk factor associated with severe complications [58]. These discrepancies may be related to the higher prevalence of bariatric surgery among women [59] and to gender roles and dynamics influencing surgical demand [60].

### Quality of life, functionality, and interpersonal relationships

The findings are consistent with studies reporting that improvements in physical functioning after bariatric surgery are associated with enhanced quality of life [61, 62]. In qualitative interviews, participants emphasized reductions in pain and respiratory problems, improvements in sleep quality, and increased physical mobility. This is consistent with literature demonstrating that improved sleep quality in individuals with obesity positively affects perceived stress levels, depressive symptoms, and weight management [39, 63, 64].

Improvements in social life and physical functioning during the first postoperative years have been reported to increase quality of life [39] and facilitate participation in the workforce [40]. However, qualitative findings indicated that social support and interpersonal relationships may have bidirectional effects on quality of life. While participants reported receiving support from family and partners, they also described experiencing jealousy and relational tension following weight loss. The literature indicates that family support after bariatric surgery enhances psychosocial well-being by promoting healthy eating and physical activity, and that weight loss may influence marital dynamics [41, 42].

### Strengths and limitations

One of the most important strengths of this study is its concurrent parallel mixed-method design. Through the systematic integration of quantitative and qualitative data, not only physical recovery but also psychosocial changes such as social relationships, emotional health, and quality of life after bariatric surgery were comprehensively and in-depth analyzed. This integrative approach contributed to a better understanding of the multidimensional nature of the postoperative experience and provided contextual and experiential insights into the relationship between personality functioning and subjective quality of life.

However, the study has several limitations. Conducting the research at a single center may limit the generalizability of the findings to different clinical settings and populations. The cross-sectional design and the focus solely on the first year following bariatric surgery also constitute important limitations. The first year is described in the literature as a “honeymoon period,” during which increased motivation and positive affect accompanying weight loss may be observed [65, 66]. Therefore, higher scores on self-report measures such as quality of life may not fully reflect long-term psychosocial adjustment. In addition, conducting only a single interview with each participant during the qualitative phase precluded the examination of potential changes over time in psychosocial processes and personality functioning. Furthermore, because the study sample consisted of patients who attended routine follow-up visits, it may have disproportionately represented individuals who were more adherent to treatment recommendations or who achieved more favorable weight-loss outcomes. Previous studies have shown that regular attendance at follow-up visits is associated with better weight-loss outcomes [67, 68]. Given the documented association between quality of life and weight loss [69], the higher quality-of-life scores observed in this study may, at least in part, reflect differences in weight-loss outcomes rather than solely the psychological variables examined. Consequently, individuals with higher level of quality-of-life may have been overrepresented in the sample compared with the broader population of patients undergoing bariatric surgery. Therefore, caution is warranted when generalizing the findings to all individuals who undergo bariatric surgery.

Another important limitation concerns the lack of systematic presentation of certain fundamental clinical and preoperative data related to sampling. Objective indicators of surgical success, such as changes in BMI, percentage of excess weight loss (%EWL), and percentage of total weight loss (%TWL), could not be obtained in a standardized, complete, and comparable manner for all participants due to the retrospective record-based data collection process. This limits the evaluation of potential relationships between psychological variables and quantitative indicators of weight loss. Similarly, detailed information regarding preoperative psychiatric history and clinically meaningful variables relevant to the tested hypotheses (e.g., presence of past psychiatric symptoms, symptom severity, previous treatment history) was not systematically reported. Although individuals with active psychiatric diagnoses were excluded during sampling, the presence of subclinical symptoms or prior psychiatric burden was not assessed. Therefore, caution should be exercised when generalizing the findings to more psychiatrically heterogeneous populations. Furthermore, because preoperative and postoperative BMI, percentage of excess weight loss, and other objective clinical indicators could not be obtained in a standardized manner for all participants, the relative contributions of psychological variables to subjective quality of life and objective surgical outcomes could not be evaluated separately. Therefore, the findings should be interpreted within the context of the associations between self-reported psychological variables and self-reported quality of life. Another important limitation is that objective clinical data regarding postoperative complications were not assessed. Consequently, it remains unclear whether scores obtained from the PBSQLS–Complications subscale reflect clinically verified complications, symptom burden, or individuals’ subjective evaluations of their postoperative experiences. Therefore, findings related to complications should be interpreted within the context of self-reported assessments.

In quantitative models, only gender and postoperative follow-up time were included as covariates. While these variables represent theoretically and statistically reasonable starting points, other clinical and behavioral confounding variables that may influence postoperative outcomes (such as type of intervention, medical comorbidities, pre- and postoperative BMI, objective weight loss indicators, psychiatric history, dietary adherence, and physical activity levels) were not included in the analyses. This suggests that the observed associations between self-reported personality functioning and postoperative outcomes may have been partially influenced by unmeasured clinical factors.

Another limitation of the study is the relatively small size of the quantitative sample (*n* = 95) and the reliance on self-report measures to assess the quantitative variables. Given the relatively small sample size and the multidimensional nature of the psychological constructs examined in this study—including personality functioning, emotion regulation difficulties, self-reported scores on the PBSQLS–Complications subscale, and quality of life—the findings should be interpreted with caution. In addition, the use of a single data collection method for all variables may have increased the risk of bias arising from common method variance and shared response tendencies. Therefore, the findings should be regarded as relational and exploratory in nature.

Furthermore, personality functioning was assessed using the self-report IPO-TR. From a psychodynamic perspective, personality organization is a complex and multidimensional construct that can be more comprehensively evaluated through structured clinical interviews, expert assessments, and longitudinal observations. Therefore, the interpretations presented in this study should not be considered as reflecting clinical levels of personality organization but rather should be understood within the context of self-reported indicators of personality functioning as assessed by the IPO-TR.

Nevertheless, the primary aim of this study was not to examine the biomedical outcomes of bariatric surgery. Rather, it was to investigate the roles of personality functioning and emotion regulation processes in quality of life following bariatric surgery and to integrate patients’ subjective experiences through a mixed-methods approach. Accordingly, the exclusion of individuals with active psychiatric diagnoses and significant chronic illnesses during sampling was a deliberate methodological choice aimed at examining the relationship between psychological variables and quality of life within a more homogeneous sample. While this approach strengthened internal validity, it may limit generalizability to patient groups with more complex clinical profiles.

Future studies should prospectively and systematically collect preoperative clinical variables and objective weight loss indicators, and employ longitudinal designs that evaluate psychological, behavioral, and biomedical variables together. Such designs would allow for a clearer understanding of the specific contribution of psychological factors to both subjective quality of life and objective surgical outcomes.

### Theoretical and practical implications

This study contributes to the growing literature on the associations of psychological factors and self-reported personality functioning with quality of life following bariatric surgery. By employing a mixed-methods design, quality of life, personality functioning, and emotion regulation difficulties were examined together, and quantitative findings were integrated with qualitative patient experiences to provide a more comprehensive understanding of these relationships.

The findings suggest that incorporating psychosocial assessment into postoperative care may be beneficial and that personality functioning may represent one of the factors that contributes to understanding patients’ postoperative experiences. Although the findings do not establish causal relationships, they highlight the importance of considering psychosocial factors during postoperative follow-up.

From a nursing perspective, the findings emphasize the importance of integrating psychological health assessments into postoperative bariatric care. Regular monitoring of emotional changes, early identification of potentially risky psychological symptoms, and referral to multidisciplinary support when necessary are essential. A patient-centered and holistic care approach may contribute to supporting long-term well-being.

Future research should examine the role of personality functioning across different cultural contexts, incorporate preoperative psychological variables into the assessment, and employ longitudinal designs to investigate changes over time. Additionally, the effectiveness of structured support programs targeting specific groups—such as women, single patients, individuals with personality disorder traits, and patients in the early follow-up period—should be tested in further studies.

## Supplementary Information

Supplementary Material 1.

## Data Availability

The datasets used and/or analysed during the current study are available from the corresponding author on reasonable request.
