# Supplementary material for: “Am I doing everything right enough?”: a mixed methods evaluation of the early post-bariatric surgery period
Source: BMC Psychol. 2026 Aug 6;14:1586. doi: 10.1186/s40359-026-05225-7 (PMC13729572; doi:10.1186/s40359-026-05225-7)
Supplement: Supplementary file 1 [file 40359_2026_5225_MOESM1_ESM.docx]

**Table 1** Trustworthiness criteria

| Criteria | **Techniques used** |
| --- | --- |
| Credibility | - ***Researcher triangulation:*** All interviews were transcribed verbatim by the second author (NSB). The transcripts were independently analyzed by two researchers (SKG and NSB). Following coding, comparison meetings were held to discuss discrepancies, and a consensus-based coding framework was established. The developed themes were also evaluated from clinical and psychosocial perspectives in team meetings that included GD and DA. - ***Member checking:*** After the preliminary themes were generated (Phase 4 of the thematic analysis process), the transcripts were sent to participants via email. They were asked to review the content for accuracy and to report any corrections, additions, or objections they deemed necessary. Thirteen out of 18 participants (72.2%) provided written feedback within two weeks. No corrections or objections regarding the content or interpretations were reported. |
| Transferability | - Participant characteristics, sampling strategies, data collection procedures, stages of analysis, and the integration of quantitative and qualitative data were described in detail to enable contextual evaluation of the findings. |
| Dependability | - ***Audit by an external researcher:*** The research design, data collection and analysis processes, and interpretation of the findings were evaluated by an independent researcher with expertise in social sciences and experience in mixed methods research. |
| Confirmability | - Methodological triangulation was applied in the data collection and analysis processes through researcher triangulation. Analytical decisions were discussed in team meetings and documented throughout the process to ensure transparency. |
